# Resequencing and Association Analysis of *PTPRA*, a Possible Susceptibility Gene for Schizophrenia and Autism Spectrum Disorders

**DOI:** 10.1371/journal.pone.0112531

**Published:** 2014-11-13

**Authors:** Jingrui Xing, Chenyao Wang, Hiroki Kimura, Yuto Takasaki, Shohko Kunimoto, Akira Yoshimi, Yukako Nakamura, Takayoshi Koide, Masahiro Banno, Itaru Kushima, Yota Uno, Takashi Okada, Branko Aleksic, Masashi Ikeda, Nakao Iwata, Norio Ozaki

**Affiliations:** 1 Department of Psychiatry, Nagoya University Graduate School of Medicine, Nagoya, Japan; 2 Department of Psychiatry, School of Medicine, Fujita Health University, Toyoake, Aichi, Japan; King Faisal Specialist Hospital and Research center, Saudi Arabia

## Abstract

**Background:**

The *PTPRA* gene, which encodes the protein RPTP-α, is critical to neurodevelopment. Previous linkage studies, genome-wide association studies, controlled expression analyses and animal models support an association with both schizophrenia and autism spectrum disorders, both of which share a substantial portion of genetic risks.

**Methods:**

We sequenced the protein-encoding areas of the *PTPRA* gene for single nucleotide polymorphisms or small insertions/deletions (InDel) in 382 schizophrenia patients. To validate their association with the disorders, rare (minor allele frequency <1%), missense mutations as well as one InDel in the 3′UTR region were then genotyped in another independent sample set comprising 944 schizophrenia patients, 336 autism spectrum disorders patients, and 912 healthy controls.

**Results:**

Eight rare mutations, including 3 novel variants, were identified during the mutation-screening phase. In the following association analysis, L59P, one of the two missense mutations, was only observed among patients of schizophrenia. Additionally, a novel duplication in the 3′UTR region, 174620_174623dupTGAT, was predicted to be located within a Musashi Binding Element.

**Major Conclusions:**

No evidence was seen for the association of rare, missense mutations in the *PTPRA* gene with schizophrenia or autism spectrum disorders; however, we did find some rare variants with possibly damaging effects that may increase the susceptibility of carriers to the disorders.

## Introduction

Schizophrenia (SCZ) is a genetically heterogeneous disorder with heritability estimated at up to 80% [1]. In recent years, although research projects such as large-scale genome-wide association studies (GWAS) have focused on common variants, they have failed to explain the majority of the heritability of SCZ [2], [3]. Subsequently, great interest has been drawn to rare (minor allele frequency, MAF <1%) missense mutations as potentially important contributing factors to the ‘missing heritability’ [4], [5]. The concept of Autism Spectrum Disorders (ASD) has been defined in the newly released Diagnostic and Statistical Manual of Mental Disorders version 5 (DSM-5) to include previous diagnoses of autistic disorder, Asperger's syndrome and PDD-NOS (pervasive developmental disorders not otherwise specified) [6]. Both SCZ and ASD are recognized as neurodevelopmental disorders, and are reported to have a major overlap of genetic risk, especially from *de novo*, deleterious mutations, [7]–[10]although further research concerning implicated loci and/or genetic risk factors (i.e., copy number variants [CNV], insertion/deletions, and single nucleotide variants) is required.

The human protein tyrosine phosphatase receptor type A (*PTPRA*) gene encodes the enzyme receptor-type tyrosine-protein phosphatase alpha (RPTP-α), a member of the protein tyrosine phosphatase (PTP) family that is involved in numerous neurodevelopmental processes related to the pathogenesis of SCZ and ASD such as myelination, radial neuronal migration, cortical cytoarchitecture formation and oligodendrocyte differentiation [11]–[14]. Moreover, RPTP-α is also functionally involved in the *neuregulin 1* (*NRG1*) signaling pathway, which regulates neurodevelopment as well as glutamatergic and gamma-aminobutyric acid–ergic neurotransmission [15]–[17]. The *NRG1* gene, together with two other genes in the same pathway—*ERBB4*, which encodes a downstream tyrosine kinase receptor[16]–[18], and *PTPRZ1*, which encodes an *ERBB4*-associated protein tyrosine phosphatase[19]—have been reported by some studies to be associated with SCZ [20]–[22].

Multiple lines of biological evidence implicate the *PTPRA* gene in the etiology of SCZ or ASD. Previous linkage studies conducted in 270 Irish high-density families (p = .0382) and an inbred, Arab Israeli pedigree of 24 members (LOD score  = 2.56 at 9.53 cM) have pointed to the area that harbors the gene [23], [24]. A GWAS comprising 575 cases and 564 controls of the Japanese ethnicity showed an association between polymorphisms within the *PTPRA* gene and SCZ (best uncorrected p = .002), albeit not at the level of genome-wide significance [25]. This result was followed by a replication study of 850 cases and 829 controls, which further confirmed the association (p = .04, p = .0008 for pooled analysis of first and second stages) [26]. Patients carrying copy number variations (CNVs) within the gene have been reported to suffer from autism, or have delayed language and speech development or stereotypical behaviors [27]. Reduced *PTPRA* expression levels have been observed in postmortem brains from patients with SCZ when compared to brains from healthy controls (13% decrease; p = .018). In the same study, a significant difference in the expression of mRNA levels of one alternative splicing variant within the gene (p = .024) was discovered in an expression analysis using lymphoblastoid cell lines (LCL) derived from 28 patients with SCZ and 20 healthy controls [26]. *Ptpra* knockout mice have been shown to exhibit neurodevelopmental deficiencies and schizophrenic-like behavioral patterns that are thought to model certain aspects of the disorder in humans. In addition, loss of *Ptpra* function in mice also leads to reduced expression of multiple myelination genes, [26] a phenomenon commonly associated with SCZ [28]–[32] and ASD [33]–[35] in human patients.

Given the aforementioned studies suggesting the association between *PTPRA* and SCZ/ASD, we decided to sequence the exonic areas of the gene in search for rare, protein-altering mutations that may further strengthen the evidence implicating *PTPRA* as a risk gene for these neurodevelopmental disorders.

## Materials and Methods

### Participants

Two independent sample sets were used in this study (Table 1). The first set, comprising 382 SCZ patients (mean age  = 53.6±14.2; male  = 56.5%;), was sequenced for missense rare variants, including single nucleotide polymorphisms (SNPs), small InDels and splicing site variations. The second, larger set, comprising 944 SCZ patients (mean age  = 50.4±15.6, male  = 58.7%), 336 ASD patients (mean age  = 19.3±10.0, male  = 77.1%), and 912 controls (mean age  = 39.1±15.9, male  = 44.5%), was used for association analysis of variants detected in the first phase.

**Table 1:** Profiles of participants in the resequencing and association sample sets.

	Sequencing	Association Study				Total
	Schizophrenia	Schizophrenia	ASD	Control	Total	
Total	382	944	336	912	2192	2574
Male	216 (56.5%)	554 (58.7%)	259 (77.1%)	406 (44.5%)	1037 (47.3%)	1253 (48.7%)
Female	166 (43.5%)	369 (39.1%)	77 (22.9%)	503 (55.2%)	1131 (51.6%)	1297 (50.4%)
Mean Age (years)	53.6±14.2	50.4±15.6	19.3±10.0	39.1±15.9	44.9±18.7	42.3±18.7

Note: Some samples in the association study group were not identified by sex.

All participants in this study were recruited in the Nagoya University Hospital and its associated institutes. Patients were included in the study if they (1) met DSM-5 criteria for SCZ or ASD and (2) were physically healthy. Controls were selected from the general population and had no personal or family history of psychiatric disorders (first-degree relatives only based on the subject′s interview). The selection was based on the following: (1) questionnaire responses from the subjects themselves during the sample inclusion step; or (2) an unstructured diagnostic interview conducted by an experienced psychiatrist during the blood collection step. All subjects were unrelated, living in the central area of the Honshu island of Japan, and self-identified as members of the Japanese population. The Ethics Committees of the Nagoya University Graduate School of Medicine approved this study. Written informed consent was obtained from all participants. In addition, the patients′ capacity to consent was confirmed by a family member when needed. Individuals with a legal measure of reduced capacity were excluded.

### Resequencing and Data Analysis

**Figure 1:**
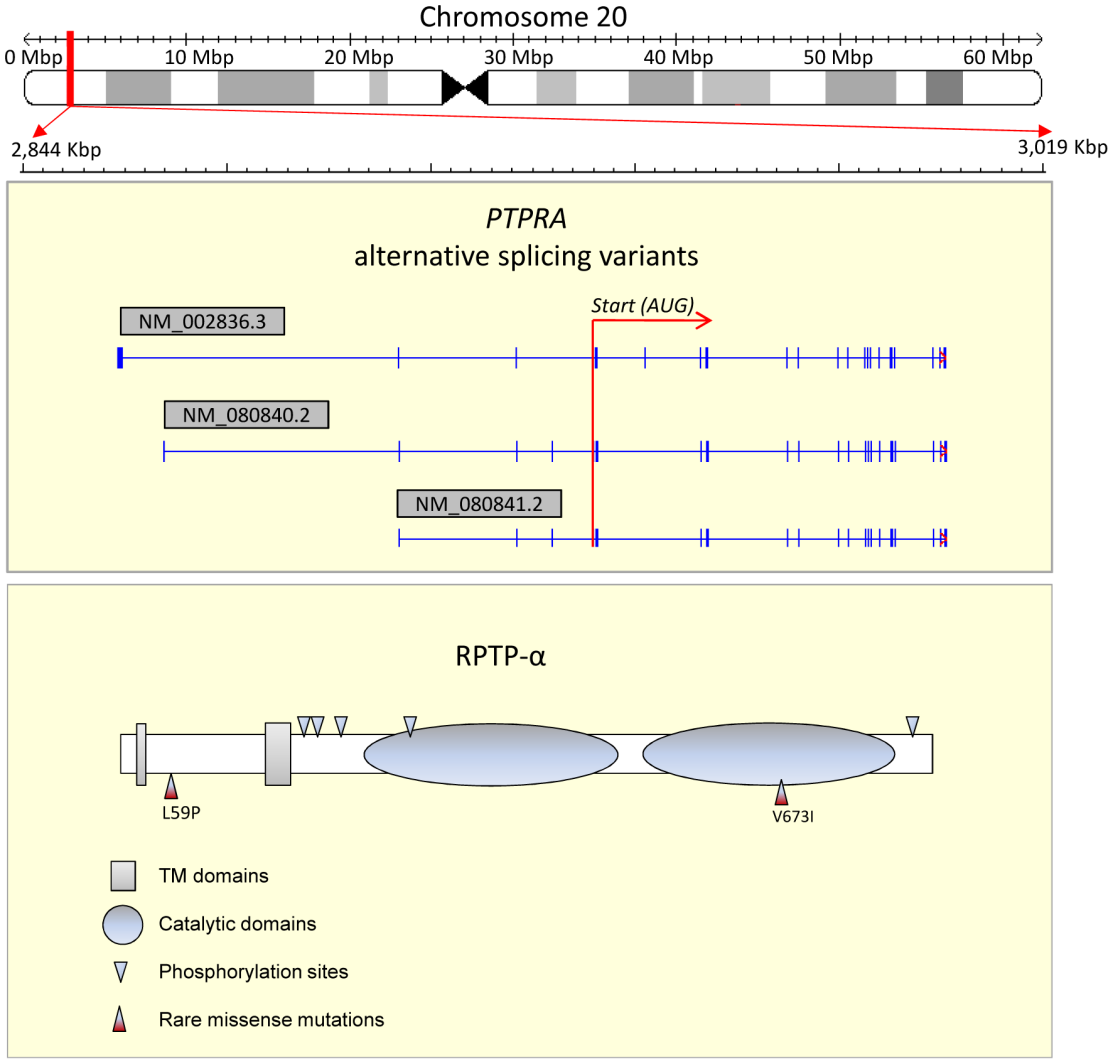
Structure of the *PTPRA* gene, RPTP-α, and position of discovered rare missense mutations.

**Figure 2:**
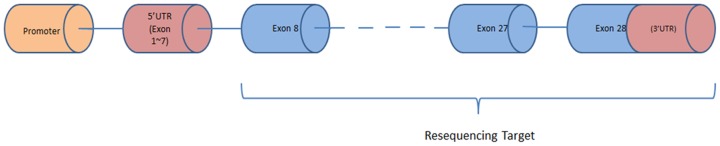
Targeted sequencing areas of the *PTPRA* Gene.

The human *PTPRA* gene is located at Chromosome 20: 2,844,830–3,019,320 and has a total of 28 exons (Ensembl release 73; Genome assembly: GRCh37; Transcript: ENST00000380393) (Fig. 1). We included only coding regions and 3′UTR (exons 8–28) (Fig. 2). Genomic DNA was extracted from whole blood or saliva using QIAGEN QIAamp DNA blood kit or tissue kit (QIAGEN Ltd. Hilden, Germany). Primers for 10 amplicons ranging from lengths of 700 to 3000 bps covering all the target exons were designed with the Primer-BLAST tool by NCBI (http://www.ncbi.nlm.nih.gov/tools/primer-blast/) and tested for validity with UCSC In-Silico PCR (http://genome.ucsc.edu/cgi-bin/hgPcr). The Takara LA taq Kit (Takara Bio Inc. Shiga, Japan) was used for PCR amplification, and products were cleaned up with Illustra Exonuclease I and Alkaline Phosphatase (GE Healthcare & Life Science, Little Chalfont, United Kingdom). After that, Sanger sequencing was performed using the BigDye Terminator v3.1 Cycle Sequencing Kit (Applied Biosystems, Foster City, California, United States). Upon the initial discovery, for all variants, we used Sanger sequencing to confirm the detection. Sequenced samples were read on an Applied Biosystems 3130xL Genetic Analyzer. Mutation detection was performed with Mutation Surveyor (Softgenetics, State College, PA, USA). The mutation calls were then revalidated for confidence.

### Association Analysis

Missense and 3′UTR mutations with MAF<1% were picked up for the association stage. Due to the altering effects that splice site variants have on the structure of mRNAs, and consequently the production of the protein, [36], [37] they were also included in the association analysis if they met the MAF criteria.

Custom TaqMan SNP genotyping assays were designed and ordered from Applied Biosystems. Allelic discrimination analysis was performed on an ABI PRISM 7900HT Sequence Detection System (Applied Biosystems, Foster City, California, United States). Differences in allele and genotype frequencies of the mutations were compared between SCZ patients/controls and ASD patients/controls using Fisher′s exact test (one-tail), with a threshold of significance set at p<0.05.

## Results

### Mutation Screening Step

Eight rare mutations consisting of 2 missense SNPs, 4 synonymous SNPs and 2 variations located in the 3′UTR area were identified within the target exons (Table 2), 4 of which were not previously reported in dbSNP Build 139 (http://www.ncbi.nlm.nih.gov/projects/SNP/), the 1000 Genomes Project (http://www.1000genomes.org), or the NHLBI Exome Sequencing Project (ESP) Variant Server (http://evs.gs.washington.edu/EVS/). All detected mutations were heterozygous.

**Table 2:** Rare exonic mutations identified during the resequencing stage.

Genomic Position a	Exon	Base Pair Change b	AA Change c	Frequency	dbSNP Reference	1000 Genomes	ESP Variant Server
20:3016327	Exon 25	171999G>GA	673V>VI	1/382	rs61742029	Registered	Registered
20:2945609	Exon 9	107281T>TC	59L>LP	2/382	Not Registered	Not Registered	Not Registered
20:3018948	3′UTR	174620_174623het_dupTGAT	—	1/382	Not Registered	Not Registered	Not Registered
20:3019013	3'UTR	174685A>AT	—	2/382	Not Registered	Not Registered	Not Registered
20:2945649	Exon 9	124753A>AG	Synonymous	4/382	rs138210276	Registered	Registered
20:3005207	Exon 21	160879G>GA	Synonymous	1/382	rs150908061	Registered	Registered
20:3017902	Exon 27	173574G>GT	Synonymous	2/382	rs375917163	Not Registered	Not Registered
20:3017903	Exon 27	173575C>CA	Synonymous	2/382	Not Registered	Not Registered	Not Registered

Notes:

a : Based on NCBI build 37.1.

b : Based on NCBI Reference Sequence NC_000020.10.

c : Based on NCBI Reference Sequence NP_001099043. AA: amino acid.

All mutations are heterozygous.

### Association Analysis

Two missense mutations, rs61742029, which had been previously observed only in the Han Chinese population, L59P, a novel variant, as well as the 174620_174623dupTGAT mutation were validated for association with SCZ and/or ASD in stage 2 (Table 3). Although we were unable to detect significance with our sample sets, it is worth noting that L59P was only present in the SCZ patient group.

**Table 3:** Association analysis results of two rare missense mutations and one 3'UTR variant.

Mutation	Genotype Counts (Resequencing) a	Genotype Counts (Association)			P Value b	
		SZ	ASD	Ctrl	SZ	ASD
171999G>GA, 673V>VI	0/3/379	0/2/942	0/2/334	0/4/908	0.3276	0.2829
101281T>TC, 59L>L/P	0/2/380	0/0/944	0/0/336	0/0/912	1.0000	1.0000
174620_174623het_dupTGAT	0/1/381	0/0/944	0/0/336	0/1/911	0.4914	1.0000

Notes:

a : Homozygote of minor allele/heterozygote/homozygote of major allele.

b : Calculated using Fisher′s exact test, one-tailed.

Ctrl: healthy controls.

### Evolutionary Conservation Analysis

Conservation status of rs61742029 and L59P in 11 common species was investigated using Mutation Taster (http://www.mutationtaster.org/). Results showed that the amino acids corresponding to the mutations in RPTP-α were highly conserved among different species (Table 4).

**Table 4:** Evolutionary conservation information for rs61742029 and L59P

Mutation	Species	Match	Gene	AA	Alignment																							
L59P	Human	—	ENST00000380393	59	K	T	S	N	P	T	S	S	L	T	S	<$>\vskip-3\raster="rg1"<$>	S	V	A	P	T	F	S	P	N	I	T	L
	Mutant	Not conserved	—	59	K	T	S	N	P	T	S	S	L	T	S	*P	S	V	A	P	T	F	S	P	N	I	T	
	P. Troglodytes	All identical	ENSPTRG00000033879	59	K	T	S	N	P	T	S	S	L	T	S	<$>\vskip-3\raster="rg1"<$>	S	V	A	P	T	F	S	P	N	I	T	
	M. Mulatta	All identical	ENSMMUG00000005878	59	K	T	S	N	P	T	S	S	L	T	S	<$>\vskip-3\raster="rg1"<$>	S	V	A	P	T	F	S	P	N	I	T	
	F. Catus	All identical	ENSFCAG00000019232	59	K	T	S	S	P	A	S	S	V	T	S	<$>\vskip-3\raster="rg1"<$>	S	V	A	P	T	F	S	P	N	L	T	
	M. Musculus	All identical	ENSMUSG00000027303	59	K	T	S	N	S	T	S	S	V	I	S	<$>\vskip-3\raster="rg1"<$>	S	V	A	P	T	F	S	P	N	L	T	
	G. Gallus	All identical	ENSGALG00000015995	56												<$>\vskip-3\raster="rg1"<$>	N	V	S	-	-	-	S	P	M	T	T	
	T. Rubripes	All identical	ENSTRUG00000014770	99	P	T	P	S	P	A	S	D	G	T	L	<$>\vskip-3\raster="rg1"<$>	Q	A	D	P	N	A	T	G	R	V	L	
	D.rerio	Not conserved	ENSDARG00000001769	101	P	P	V	V	P	P	P	A	V	P	I	*P	T	V	V	L	P	V	P	P	T	P	T	
	D. Melanogaster	No homologue	—	N/A																								
	C. Elegans	No alignment	C09D8.1	N/A																								
	X. Tropicalis	All conserved	ENSXETG00000017982	71		T	T	A	PF	T	T	T	R	T	A	<$>\vskip-3\raster="rg2"<$>	I	L	A	P	N	V	T	D	S	I	F	
rs61742029	Human			664	L	K	K	E	E	E	C	E	S	Y	T	*V	R	D	L	L	V	T	N	T	R	E	N	
	mutated	all conserved		664									S	Y	T	<$>\vskip-3\raster="rg3"<$>	R	D	L	L	V	T	N	T	R	E	N	
	Ptroglodytes	all identical	ENSPTRG00000033879	673	L	K	K	E	E	E	C	E	S	Y	T	<$>\vskip-3\raster="rg4"<$>	R	D	L	L	V	T	N	T	R	E	N	
	Mmulatta	all identical	ENSMMUG00000005878	673	L	K	K	E	E	E	C	E	S	Y	T	<$>\vskip-3\raster="rg4"<$>	R	D	L	L	V	T	N	T	R	E	N	
	Fcatus	all identical	ENSFCAG00000019232	674	L	K	K	E	E	E	C	E	S	Y	T	<$>\vskip-3\raster="rg4"<$>	R	D	L	L	V	T	N	T	R	E	N	
	Mmusculus	all identical	ENSMUSG00000027303	700	L	K	K	E	E	E	C	E	S	Y	T	<$>\vskip-3\raster="rg4"<$>	R	D	L	L	V	T	N	T	R	E	N	
	Ggallus	all identical	ENSGALG00000015995	680	L	K	K	E	E	E	C	E	S	Y	T	<$>\vskip-3\raster="rg4"<$>	R	D	L	L	V	T	N	T	R	E	N	
	Trubripes	all identical	ENSTRUG00000014770	710										Y	T	<$>\vskip-3\raster="rg4"<$>	R	D	L	L	V	T	N	N	R	E	N	

*Marks the position of the amino acid change due to mutation.

### *In Silico* Functional Effects Prediction

Possible functional implications brought by amino acid changes due to the 2 missense mutations were analyzed with PolyPhen-2 (http://genetics.bwh.harvard.edu/pph2/), PMut (http://www.ngrl.org.uk/Manchester/page/pmut) and SIFT (http://sift.jcvi.org/). (Table 5) According to the results, the mutation L59P, which was only observed in schizophrenia patients, was predicted to be mostly benign, while rs61742029 showed a high probability of pathogenicity in PolyPhen-2.

**Table 5:** In silico functional effect prediction for rs61742029 and L59P.

Mutation	Prediction Tool		
	PolyPhen-2	Pmut	SIFT
rs61742029	Probably damaging	Neutral	Tolerated
L59P	Benign	Neutral	Tolerated

### 3′UTR Motif Prediction

174620_174623dupTGAT, a small duplication discovered in the 3′UTR area, was predicted by RegRNA 2.0 (http://regrna2.mbc.nctu.edu.tw) to be located within a human Musashi Binding Element (MBE), an evolutionarily conserved region shown to affect neural cell differentiation through its mRNA translation regulator properties [38].

### Clinical Information of the Carriers of Mutation L59P and 174620_174623dupTGAT

The patient carrying the *PTPRA* L59P mutation was a male diagnosed with SCZ at the age of 19. The patient was born in 1947 had a normal course of development during childhood. In early 1966, he started to suffer from auditory hallucinations, and soon withdrew into an indoor lifestyle. His family reported him being irritated when visited, as well as behaving improperly in public. He was promptly diagnosed and admitted to a psychiatry ward in the same year, and spent the rest of his life living in a hospital. A remarkable improvement was observed in his positive symptoms after admission and administration of antipsychotic drugs; however, he remained secluded, hardly communicating with people around him. At the time of his enrollment in the study, he was 162 cm tall and weighed 48 kg. No comorbid physical or mental illnesses were present. He had 3 children, among whom, one daughter had a history of mental disorder. The patient succumbed to pneumonia in the second half of 2013. In a computerized axial tomography (CAT) scan of the head taken a few weeks prior to patient′s death, diffuse neocortical atrophy was observed.

The other patient carrying the L59P mutation was a female diagnosed with SCZ at the age of 34. No childhood development abnormalities were reported, but she was noted to have a history of irritability/aggressive tendencies in high school. Since onset, she had experienced auditory hallucinations and persecutory delusions, as well as continued irritability and aggression. Despite the efficacy of antipsychotic drugs on her positive symptoms, the patient suffered numerous relapses throughout her course of illness due to poor insight and lack of adherence to treatment. At the time of recruitment, she was 61 years old, with a chronic condition of diabetes and no comorbid mental conditions. She died in 2012 at the age of 62.

The patient carrying the *PTPRA* 174620_174623dupTGAT mutation was a male diagnosed with SCZ and comorbid intellectual disability at the age of 27, while he was enrolled in our study. He had a normal conception and birth, born to a 28-year-old father and 27-year-old mother. His father died when he was 3. Delayed intellectual development was observed since his childhood, with reports of illiteracy, hyperactivity, poor concentration and low performance at school. He subsequently dropped out of high school in his first year and started attending a technical school. After graduation, not being able maintain a steady position, he changed part-time jobs frequently. He presented at onset with hallucinations, persecutory delusions, and psychomotor excitement, and was subjected to involuntary commitment due to harmful behavior to others as a result of his delusions. At the time of admission, he was 168 cm tall and weighed 74 kg, with a Wechsler Adult Intelligence Scale (WAIS) score of 49 (Verbal IQ = 57, Performance IQ = 48); he also suffered from stuttering (anarthria literalis). After remission under antipsychotic treatment, he was discharged; however, lack of insight or compliance persisted. It was reported that his mother had a history of panic attacks, and one of his maternal relatives was also diagnosed with SCZ.

## Discussion

To our knowledge, this is the first study that systematically screened all coding regions and 3′UTR of the *PTPRA* gene for rare variants in SCZ patients and assessed the association of identified mutations in such a study with SCZ/ASD.

### Main Findings

In this study, we sequenced the encoding regions, splicing sites, and 3′UTR region of the *PTPRA* gene in 382 SCZ patients using the Sanger sequencing method, and discovered 8 rare variants. We then conducted association analysis in a much larger sample set for the 2 rare, missense mutations and one 3′UTR InDel identified during the mutation-screening phase in order to investigate their relationship with SCZ and/or ASD.

We were unable to detect a statistically significant association for any of the 3 mutations; this may be attributed partially to the low frequency of rare mutations in the population. However, according to our estimation using CaTS, the power calculator for two-stage association studies (http://www.sph.umich.edu/csg/abecasis/CaTS/), it would require a sample size of around 25,000 cases and controls for the study to obtain possible significance [39], [40]. Also, L59P was only detected among SCZ patients in our sample, which infers possible connection of this mutation to the disorder. The evolutionary conservation status of the locus also indicates its biological importance.

Recent studies have discussed the limited impact of protein-coding variants detected in exome resequencing projects, attributing it partly to the fact that most associated variants alter gene expression rather than protein structure. These findings may help explain the lack of association for the 2 missense mutations we detected, while hinting that 174620_174623dupTGAT, predicted to be located within an expression-regulating element, may have a more significant effect. [10]

Additionally, an increasing amount of evidence suggests that genetic risks for SCZ and ASD may not be conferred by the effects of individual variants alone, but also the amplifying interactions between multiple susceptibility loci [41]–[44]. Thus it may be interesting to sequence the mutation carriers for additional related variants in future.

### Limitations

Several limitations should be considered when interpreting the results of our study. The single candidate gene paradigm for a gene with less than robust ties to schizophrenia may have been one of the reasons leading to negative results. Besides, the Sanger method it employed predetermined its relatively small sample size and detection power in contrast with next generation resequencing. In addition, we did not have lymphoblastoid cell lines (LCLs) from the mutation carriers for expression analysis or blood samples from their family members for pedigree study. Therefore, we were unable to follow up the results with further biological evidence. Moreover, some potentially interesting regions of the *PTPRA* gene, such as the promoter, 5′UTR, and most of the intronic areas, were not sequenced (the rare intronic mutations we detected close to the exons can be viewed in Table S1).

### Conclusion

In conclusion, our study did not detect any rare missense mutations within the *PTPRA* gene in our samples that showed statistical association with SCZ or ASD. Nonetheless, some potentially interesting variants were identified that might increase the susceptibility of their carriers to the disorders. Also, our results may help provide genetic clues for the involvement of the *PTPRA* gene in the pathogenesis of psychiatric disorders.

## Supporting Information

Table S1 Rare intronic mutations identified during the resequencing stage. ^a^: Based on NCBI build 37.1. ^b^: Based on NCBI Reference Sequence NC_000020.10. All mutations are heterozygous.
(DOCX)
