# Supplementary material for: Resequencing and Association Analysis of PTPRA, a Possible Susceptibility Gene for Schizophrenia and Autism Spectrum Disorders
Source: PLoS One. 2014 Nov 13;9(11):e112531. doi: 10.1371/journal.pone.0112531 (PMC4231042; doi:10.1371/journal.pone.0112531)
Supplement: Table S1 — Rare intronic mutations identified during the resequencing stage. a: Based on NCBI build 37.1. b: Based on NCBI Reference Sequence NC_000020.10. All mutations are heterozygous. (DOCX) [file pone.0112531.s001.docx]

**Table S1.** Rare intronic mutations identified during the resequencing stage

| \| Genomic Position ^a^ \| Position in Gene \| Mutation ^b^ \| dbSNP ID \| Frequency \| \| --- \| --- \| --- \| --- \| --- \| \| 20:2945122 \| Intron 8-9 \| 100894c>cg \| novel \| 2/382 \| \| 20:2967122 \| Intron 10-11 \| 122794t>ta \| novel \| 1/382 \| \| 20:2967606 \| Intron 10-11 \| 123278het_dela \| novel \| 1/382 \| \| 20:3001793 \| Intron 17-18 \| 157465a>ag \| novel \| 1/382 \| \| 20:3002201 \| Intron 18-19 \| 157873a>ag \| novel \| 1/382 \| \| 20:3003282-3003284 \| Intron 19-20 \| 158954_158956het_delgct \| rs3833326 \| 4/382 \| \| 20:3002925 \| Intron 19-20 \| 158597het_dela \| novel \| 1/382 \| \| 20:3003122 \| Intron 19-20 \| 158794g>ga \| novel \| 2/382 \| \| 20:3004942 \| Intron 20-21 \| 160614g>gt \| novel \| 1/382 \| \| 20:3008332 \| Intron 23-24 \| 164004c>ct \| novel \| 1/382 \| \| 20:3008601 \| Intron 23-24 \| 164273c>ct \| novel \| 1/382 \| \| 20:3016193 \| Intron 24-25 \| 164227a>at \| novel \| 1/382 \| \| 20:3016115 \| Intron 24-25 \| 171787g>ga \| novel \| 1/382 \| \| 20:3017622 \| Intron 26-27 \| 173294c>ct \| novel \| 2/382 \| |  |  |  |  |  |  |
| --- | --- | --- | --- | --- | --- | --- | --- | --- | --- | --- | --- | --- | --- | --- | --- | --- | --- | --- | --- | --- | --- | --- | --- | --- | --- | --- | --- | --- | --- | --- | --- | --- | --- | --- | --- | --- | --- | --- | --- | --- | --- | --- | --- | --- | --- | --- | --- | --- | --- | --- | --- | --- | --- | --- | --- | --- | --- | --- | --- | --- | --- | --- | --- | --- | --- | --- | --- | --- | --- | --- | --- | --- | --- | --- | --- | --- | --- | --- | --- | --- | --- |
| Notes:  ^a^: Based on NCBI build 37.1.  ^b^: Based on NCBI Reference Sequence NC_000020.10.  All mutations are heterozygous. |  |  |  |  |  |  |
